# Definitive Simultaneous Integrated Boost Versus Conventional-Fractionated Intensity Modulated Radiotherapy for Patients With Advanced Esophageal Squamous Cell Carcinoma: A Propensity Score-Matched Analysis

**DOI:** 10.3389/fonc.2021.618776

**Published:** 2021-06-21

**Authors:** Chen Li, Lijun Tan, Xiao Liu, Xin Wang, Zongmei Zhou, Dongfu Chen, Qinfu Feng, Jun Liang, Jima Lv, Xiaozhen Wang, Nan Bi, Lei Deng, Wenqing Wang, Tao Zhang, Wenjie Ni, Xiao Chang, Weiming Han, Zefen Xiao

**Affiliations:** ^1^ Department of Radiation Oncology, National Cancer Center/National Clinical Research Center for Cancer/Cancer Hospital, Chinese Academy of Medical Sciences and Peking Union Medical College, Beijing, China; ^2^ Department of Oncology, First Affiliated Hospital of Harbin Medical College, Harbin, China; ^3^ Department of Radiation Oncology, Henan Cancer Hospital, Zhengzhou, China; ^4^ Department of Radiation Oncology, Cancer Hospital Chinese Academy of Medical Sciences, Shenzhen Center, Shenzhen, China

**Keywords:** esophageal cancer, definitive radiotherapy, intensity-modulated radiotherapy, simultaneous integrated boost, propensity score matching

## Abstract

**Background:**

The aim of this study was to compare the effects of simultaneous integrated boost–intensity modulated radiotherapy (SIB-IMRT) and conventional fractionated-IMRT (CF-IMRT) for patients with esophageal squamous cell carcinoma (ESCC).

**Methods:**

The data of 1173 patients treated with either CF-IMRT or SIB-IMRT for a curative intent from 2005 to 2016 were retrospectively reviewed. Propensity score matching (PSM) was used to create a well-balanced cohort of 687 patients at 1:2 ratio (237 patients in SIB-IMRT group and 450 patients in CF-IMRT group). Overall survival (OS), progression-free survival (PFS), recurrence pattern, and toxicity profiles were evaluated and compared between the two groups after PSM.

**Results:**

After a median follow-up time of 42.3 months (range, 3.0-153.2 months) for surviving patients, survival results were comparable in the two groups. After PSM, the 1-year, 2-year and 4-year OS rates in the SIB-IMRT and CF-IMRT groups were 70.0% *vs.* 66.4%, 41.9% *vs.* 41.7% and 30.2% *vs.* 27.6%, respectively (*p* = 0.87). The 1-year, 2-year and 4-year PFS rates were 48.4% *vs.* 49.1%, 31.2% *vs.* 29.4%, and 26.1% *vs.* 17.9%, respectively (*p* = 0.64). Locoregional recurrence (*p* = 0.32) and distant metastasis (*p* = 0.54) rates were also comparable between two groups. The toxicity profile was similar in the two groups. Multivariate analyses in the matched samples showed that female, concurrent chemotherapy and earlier clinical stage were independently associated with longer OS and PFS.

**Conclusions:**

SIB-IMRT appears to be equivalent to CF-IMRT in treatment efficacy and safety, and could become an alternative option for definitive radiotherapy of ESCC.

## Introduction

Esophageal cancer is a common malignancy with poor prognosis, according to a recent epidemiological survey, it is the third most common cancer and the fourth leading cause of cancer death in China (1). Due to insufficient promotion of annual endoscopic screening, a large proportion of patients in China have advanced disease at diagnosis. These patients are not suitable for esophagectomy and are usually treated with definitive radiotherapy plus concurrent chemotherapy (2, 3). For this group of patients, most guidelines nowadays recommended concurrent chemoradiotherapy with a radiation dose of 50.4 Gy (4, 5). However, recent research showed poor local control with this radiation dose (6). In most Asian countries, 60 Gy was a more commonly used radiation dose (7), however, to achieve this dose without significantly increasing the incidence of toxicities, a technique called “sequential boost” was commonly used before the emergence of IMRT, which requires re-simulation and re-planning in one treatment course.

Developed in the 1990s, intensity-modulated radiotherapy (IMRT), was a major breakthrough in radiotherapy technique (8). With the ability to modulate the intensity of each beam and computer-controlled multi-leaf collimation, IMRT greatly improved treatment conformality and sparing of normal structures. According to Lin et al, ﻿esophageal cancer patients treated with conventional fractionated-IMRT (CF-IMRT) had a lower risk of mortality and of locoregional recurrence compared to those treated with three-dimensional conformal radiotherapy (3DCRT) (9). In the early 2000s, radiation oncologists further exploited the ability of the IMRT technique to deliver heterogeneous doses and created the simultaneous integrated boost (SIB)-IMRT technique, with which it became possible to administer different doses to different regions within one treatment fraction (10–12). SIB-IMRT has since been used to treat different types of malignancies and has gradually become a norm in the treatment of many solid tumors such head and neck cancer (12–16). However, due to the potential risks of serious side effects such as bleeding or perforation, SIB-IMRT has not been generally used for treatment of tumors on cavity organs (esophagus, stomach, intestine, rectum, trachea, etc.), and therefore little is known about how it compares with CF-IMRT in terms of survival, recurrence pattern, and toxicities.

At our center the CF-IMRT technique has been widely used since 2004, whereas SIB-IMRT has been gradually applied in the treatment of esophageal cancer since 2010. This study retrospectively collected data of patients treated with either technique to make a comparison between them on clinical outcomes, relevant side effects, etc. Propensity score matching (PSM) (17) was used to correct for potential selection bias and covariate imbalances between the groups.

## Materials and Methods

### Patients Selection Criteria

**Figure 1:**
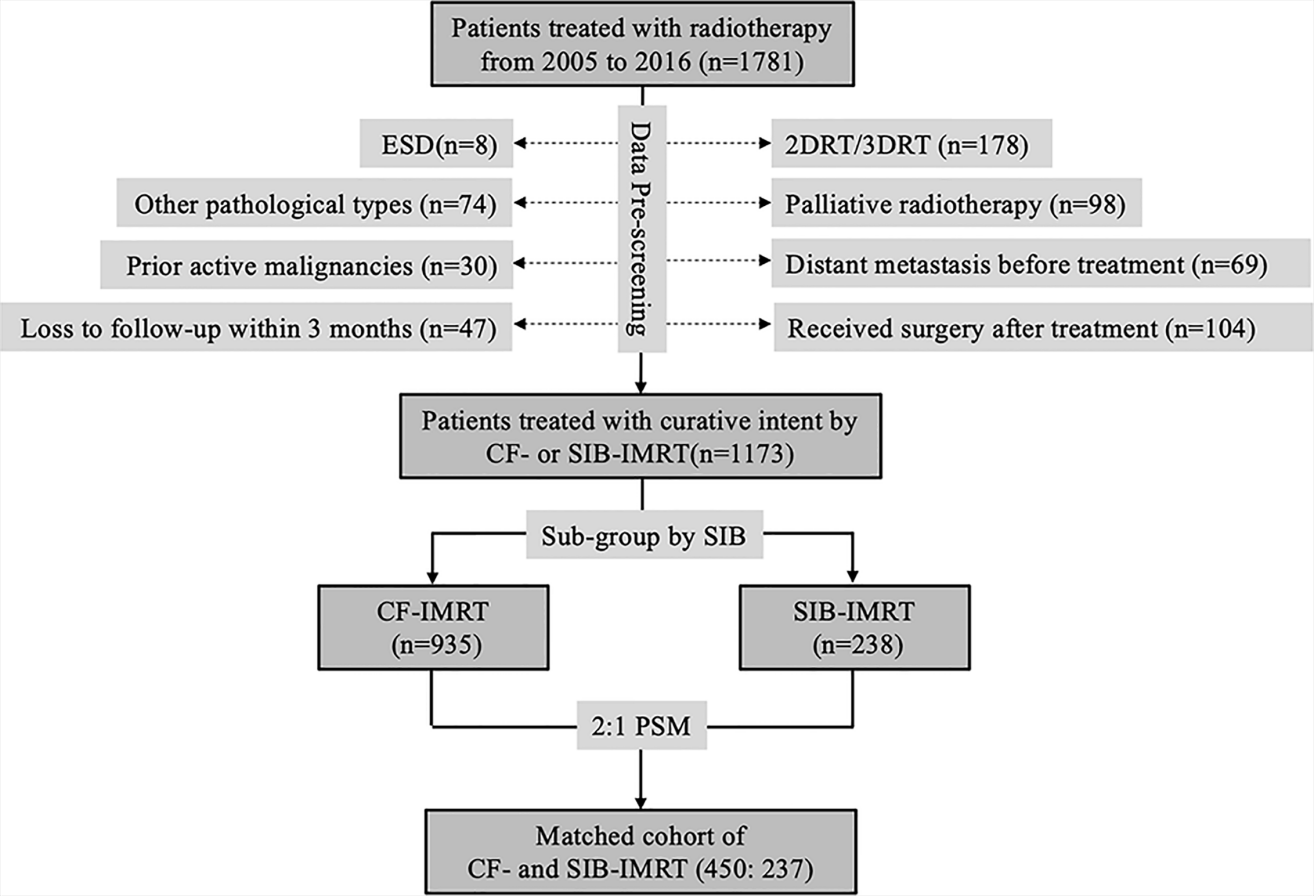
CONSORT diagram showing patient selection. ESD, endoscopic submucosal dissection; 2DRT, two-dimensional radiotherapy; 3DRT, three-dimensional radiotherapy; IMRT, intensity-modulated radiotherapy; CF-IMRT, conventional fractionation IMRT; SIB-IMRT, simultaneous integrated boost-IMRT; PSM, propensity score matching.

A total of 1781 patients with biopsy-confirmed esophageal squamous cell carcinoma (SCC) received radiotherapy with or without concurrent chemotherapy with a definitive purpose at our center between 2005 and 2016. All patients were considered as inoperable patients by a ﻿multidisciplinary team (comprising radiologists, thoracic surgeons, radiation oncologists, medical oncologists, and other specialists) for the following reasons: 1) unresectable primary tumor (with invasion of adjacent organs such as aorta, vertebrae, bronchus, and so on) or metastatic lymph nodes; 2) severe comorbidities (e.g., myocardial infarction, severe chronic obstructive pulmonary disease, insufficient pulmonary function, cerebral infarction, and so on); 3) refusal of consent by patient or relatives; or 4) advanced age or poor general health condition. Patients were excluded from this study if they had 1) distant visceral metastasis at presentation (n = 69); 2) active malignancies (other than curable non-melanoma skin cancer or *in situ* cervical cancer) within the past 5 years (n = 30); 3) received two-dimensional radiotherapy (2DRT) or 3DCRT (n = 178); 4) been diagnosed with pathological types other than SCC (e.g., adenocarcinoma, small cell carcinoma, and so on; n = 74); 5) received only palliative treatment (radiation dose < 40 Gy or palliative target area; n = 98); 6) ﻿﻿loss to follow-up within 3 months after radiotherapy (n = 47); 7) undergone endoscopic submucosal resection before radiotherapy (n = 8); or 8) had neoadjuvant radiotherapy/chemoradiotherapy and received surgery afterwards (n=104). Thus, finally, the final cohort comprised 1173 patients, of which 238 received SIB-IMRT and 935 received CF-IMRT (**Figure 1**). All patients were staged according to the 6th edition (2002) of the American Joint Committee on Cancer TNM staging (18).

### Treatment

The gross tumor volume (GTV-T) was defined as the primary tumor. The GTV-T was determined using all available resources (physical examination, upper gastrointestinal contrast study, endoscopy, endoscopic ultrasound (EUS), contrast-enhanced computed tomography [CT] of thorax/abdomen, contrast-enhanced magnetic resonance imaging [MRI] of thorax/abdomen, positron-emission tomography [PET]-CT, and so on). Metastatic regional nodes (GTV-N) referred to any lymph node confirmed or highly suspected as metastasis. Clinical Target Volume (CTV) adopted elective nodal irradiation and incorporated prophylactic lymphatic drainage regions. Planning target volume (PTV) included the CTV plus a uniform 0.5 cm margin. For patients receiving SIB-IMRT, an additional target called planning gross tumor volume (PGTV) was created by expanding GTV-T by 1.0 cm craniocaudally and 0.5 cm radially, and the GTV-N by a uniform 0.5 cm margin. The dose for patients receiving SIB-IMRT was based on the results of our previous phase I/II dose-escalation study (19); the most common prescription dose to PTV was 50.4 Gy in 28 fractions, the most common prescription dose to PGTV was 59.92 Gy in 28 fractions. For CF-IMRT patients a prophylactic irradiation of 40-50 Gy was first delivered to ≥ 95% of PTV and then, after re-simulation/treatment planning, a sequential boost of 10-20 Gy was given to the primary tumor and metastatic lymph nodes. Image-guided radiotherapy was used every day for the first week of treatment and once a week for the rest of the treatment course.

For non-elderly patients with favorable general health conditions, chemotherapy was often administered in combinations of taxane and platinum-based compounds. For elderly patients, single oral agent (e.g., S-1, capecitabine, etc.) was a more common choice. A small group of patients received induction chemotherapy prior to radiotherapy and some patients received concurrent target drug. The most commonly used concurrent target drug is Nimotuzumab.

### Outcome Measures

Acute and late toxicities were scored according to the Common Toxicity Criteria for Adverse Events, version 4.0 (20). All patients were assessed weekly during treatment, every 3 months during the first 2 years after treatment, every 6 months for the next 3 years, and annually thereafter. Evaluation included contrast-enhanced CT of neck, chest, and upper abdomen; barium esophagram; ultrasonography of the neck and upper abdomen; and conventional blood and biochemistry studies. EUS, PET-CT, and fine-needle aspiration cytology were performed when indicated. Bone scan was performed in case of bone pain or abnormal elevation of serum alkaline phosphatase. Cranial MRI was performed if indicated.

Recurrences were classified as local-regional recurrence (LRR) or distant metastases (DM). LRR was defined as any recurrence at the primary site or in regional lymph nodes. Recurrence at distant sites was recorded as DM. Multiple recurrences detected within 1 month of one another were considered synchronous.

Overall survival (OS) was measured as the interval from start of radiotherapy to the date of death from any cause or last follow-up. Progression-free survival (PFS) was defined as the interval from the start of radiotherapy to the date of detection of first recurrence, death from any cause, or last follow-up. Patients who had not experienced progression or death by the last follow-up were administratively censored. Time to recurrence of any type was calculated from the completion of radiotherapy.

### Statistical Analysis

Descriptive statistics, including frequencies and percentages for categorical variables, and median and standard deviation for quantitative variables, were computed to summarize patient characteristics for the overall patient cohort and for each treatment group. Between-group comparisons were conducted using the *t*-tests, log-rank test, and chi-square test for continuous, nominal, and ordinal variables, respectively. PSM was used to adjust for unbalanced covariates. Tumor location, disease stage and concurrent chemotherapy were included in the propensity score matching system since they were considered unbalanced between CF-IMRT and SIB-IMRT groups, among which exact matching method was performed on disease stage and concurrent chemotherapy whereas nearest matching method was adopted for tumor location. With caliper setting of 0.25 and a 1:2 allocation two comparable groups of patients were created: the SIB-IMRT group (n = 237) and the CF-IMRT group (n = 450). OS, PFS, LRR rate, and DM rate before and after PSM were estimated for each treatment group using Kaplan-Meier plots. The log-rank test was used to compare event–time distributions between the two groups. A Cox regression model was used for multivariate analyses of the effect of covariates on OS after PSM. The significance level was set as *p* ≤ 0.05. All computations were conducted using R version 2.13.0 (https://www.r-project.org/).

## Results

### Patient and Treatment Characteristics

A total of 1173 patients were included in this study: 238 in the SIB-IMRT group and 935 in the CF-IMRT group. The median follow-up time for surviving patients in the entire cohort was 42.3 months (range 3.0-153.2 months); it was 50.2 months (range 3.0-153.2 months) for the CF-IMRT group and 30.9 months (range 4.5-91.0 months) for the SIB-IMRT group. **Table 1** summarizes the patient, tumor, and treatment characteristics in patients before and after PSM. As shown in the table, most patients enrolled in this study had locally advanced disease. More than 40% of patients had a primary tumor of T4, and more than 80% of patients were stage III-IV. For patients receiving CF-IMRT, the most commonly used fractionation regimen was a daily fraction of 2.0 Gy to a total dose of 60 Gy. For patients receiving SIB-IMRT, the most commonly used fractionation regimen was a daily fraction of 2.14 Gy to PGTV to a total dose of 59.92 Gy (with a daily fraction of 1.8 Gy to PTV to a total dose of 50.4 Gy). Due to the slight difference in the dose fractionation regimen of the enrolled patients, the total dose was also converted into equivalent dose in 2 Gy/fraction (EQD2) using an α/β of 10 and presented in **Table 1**. SIB-IMRT patients were more likely to have advanced disease (stage IV) than CF-IMRT patients (32.8% *vs.* 26.5%; *p* = 0.01); to have middle and lower thoracic tumors (middle esophagus: 52.9% *vs.* 46.5%; lower esophagus: 23.5% *vs.* 17.2%; *p* = 0.001); to have received concurrent chemotherapy (56.3% vs. 44.1%; *p* < 0.001); and to be diagnosed at a more recent time (diagnosed between 2011 and 2016: 90.3% *vs.* 67.1%; *p* < 0.001).

**Table 1:** Patient, tumor, and treatment characteristics, before and after propensity score matching.

	Overall		Before Matching				After Matching					
	n = 1173 (%)		CF-IMRT		SIB-IMRT		*p*	CF-IMRT		SIB-IMRT		*p*
			n = 935 (%)		n = 238 (%)			n = 450 (%)		n = 237 (%)		
Age							0.16					0.71
Median (Range)	63 (33-89)		63 (33-89)		62 (43-84)			61 (33-84)		62 (43-84)		
Sex							0.62					0.49
Male	952 (81.2)		762 (81.5)		190 (79.8)			370 (82.2)		189 (79.7)		
Female	221 (18.8)		173 (18.5)		48 (20.2)			80 (17.9)		48 (20.3)		
KPS							0.70					0.71
60-70	117 (10.0)		96 (10.3)		21 (8.8)			39 (8.7)		21 (8.9)		
80	681 (58.1)		541 (57.9)		140 (58.8)			260 (57.8)		140 (59.1)		
90-100	375 (32.0)		298 (31.9)		77 (32.4)			151 (33.6)		76 (32.1)		
T stage (6th)							0.75					0.98
T1	41 (3.5)		34 (3.6)		7 (2.9)			13 (2.9)		6 (2.8)		
T2	119 (10.1)		100 (10.7)		19 (8.0)			37 (8.2)		19 (8.0)		
T3	491 (41.9)		388 (41.5)		103 (43.3)			184 (40.9)		103 (43.5)		
T4	518 (44.2)		410 (43.9)		108 (45.4)			214 (47.6)		108 (45.6)		
Unknown	4 (0.3)		3 (0.3)		1 (0.4)			2 (0.4)		1 (0.4)		
N stage (6th)							<0.01					0.06
N0	169 (14.4)		153 (16.4)		16 (6.7)			55 (12.2)		16 (6.8)		
N1	1001 (85.3)		779 (83.3)		222 (93.3)			394 (87.6)		221 (93.2)		
Unknown	3 (0.3)		3 (0.3)		0 (0.0)			1 (0.2)		0 (0.0)		
M stage (6th)							<0.01					0.34
M0	845 (72.0)		686 (73.4)		159 (66.8)			303 (67.3)		158 (66.7)		
M1a	119 (10.1)		99 (10.6)		20 (8.4)			49 (10.9)		20 (8.4)		
M1b	208 (17.7)		150 (16.0)		58 (24.4)			98 (21.8)		58 (24.5)		
Unknown	1 (0.1)		0 (0.0)		1 (0.4)			0 (0.0)		1 (0.4)		
TNM stage (6th)							0.01					0.98
0-II	172 (14.7)		148 (15.8)		24 (10.1)			43 (9.6)		23 (9.7)		
III	675 (57.5)		539 (57.6)		136 (57.1)			260 (57.8)		136 (57.4)		
IV	326 (27.8)		248 (26.5)		78 (32.8)			147 (32.7)		78 (32.9)		
Tumor location							<0.01					0.19
Cervical	49 (4.2)		40 (4.3)		9 (3.8)			22 (4.9)		9 (3.8)		
Upper	346 (29.5)		299 (32.0)		47 (19.7)			94 (20.9)		47 (19.8)		
Middle	561 (47.8)		435 (46.5)		126 (52.9)			251 (55.8)		126 (53.2)		
Lower	217 (18.5)		161 (17.2)		56 (23.5)			83 (18.4)		55 (23.2)		
Radiation dose (Gy)							0.76					0.29
Median (Range)	60 (40-72)		60 (40-70)		59.92 (40-72)			60 (40-70)		59.92 (40-72)		
Fractionation size(Gy)												
Median (Range)			2.0 (1.8-2.2)		2.14 (2.0-2.4)			2.0 (1.8-2.0)		2.14 (2.0-2.4)		
EQD2 (Gy)												
Median (Range)	60 (40-72)		60 (40-70)		60.6 (40-72)			60 (40-70)		60.6 (40-72)		
Concurrent chemotherapy							<0.01					0.61
No	627 (53.5)		523 (55.9)		104 (43.7)			208 (46.2)		104 (43.9)		
Yes	546 (46.5)		412 (44.1)		134 (56.3)			242 (53.8)		133 (56.1)		
Induction chemotherapy							0.83					0.66
No	1098 (93.6)		874 (93.5)		224 (94.1)			418 (92.9)		223 (94.1)		
Yes	75 (6.4)		61 (6.5)		14 (5.9)			32 (7.1)		14 (5.9)		
Concurrent target drug							0.92					0.52
No	1069 (91.1)		853 (91.2)		216 (90.8)			416 (92.4)		215 (90.7)		
Yes	104 (8.9)		82 (8.8)		22 (9.2)			34 (7.6)		22 (9.3)		
Year of diagnosis							<0.01					0.94
2005-2010	331 (28.2)		308 (32.9)		23 (9.7)			46 (10.2)		23 (9.7)		
2011-2016	842 (71.8)		627 (67.1)		215 (90.3)			404 (89.8)		214 (90.3)		

CF-IMRT, conventional fractionated-intensity modulated radiotherapy; SIB-IMRT, simultaneous integrated boost-intensity modulated radiotherapy; KPS, Karnofsky Performance Status; EQD2, equivalent dose in 2 Gy/fraction.

### Survival Results

No differences were seen in OS and PFS between the SIB-IMRT group and the CF-IMRT group in the entire before-match cohort. Median OS was 19.2 months (95% CI: 15.9-22.4 months) in the SIB-IMRT group *vs.* 20.2 months (95% CI: 18.5-22.0 months) in the CF-IMRT group (log-rank *p* = 0.71). Median PFS was 11.3 months (95% CI: 9.0-13.7 months) in the SIB-IMRT group and *vs.* 13.1 months (95% CI: 11.7-14.5 months) in the CF-IMRT group (log-rank *p* = 0.61).

**Figure 2:**
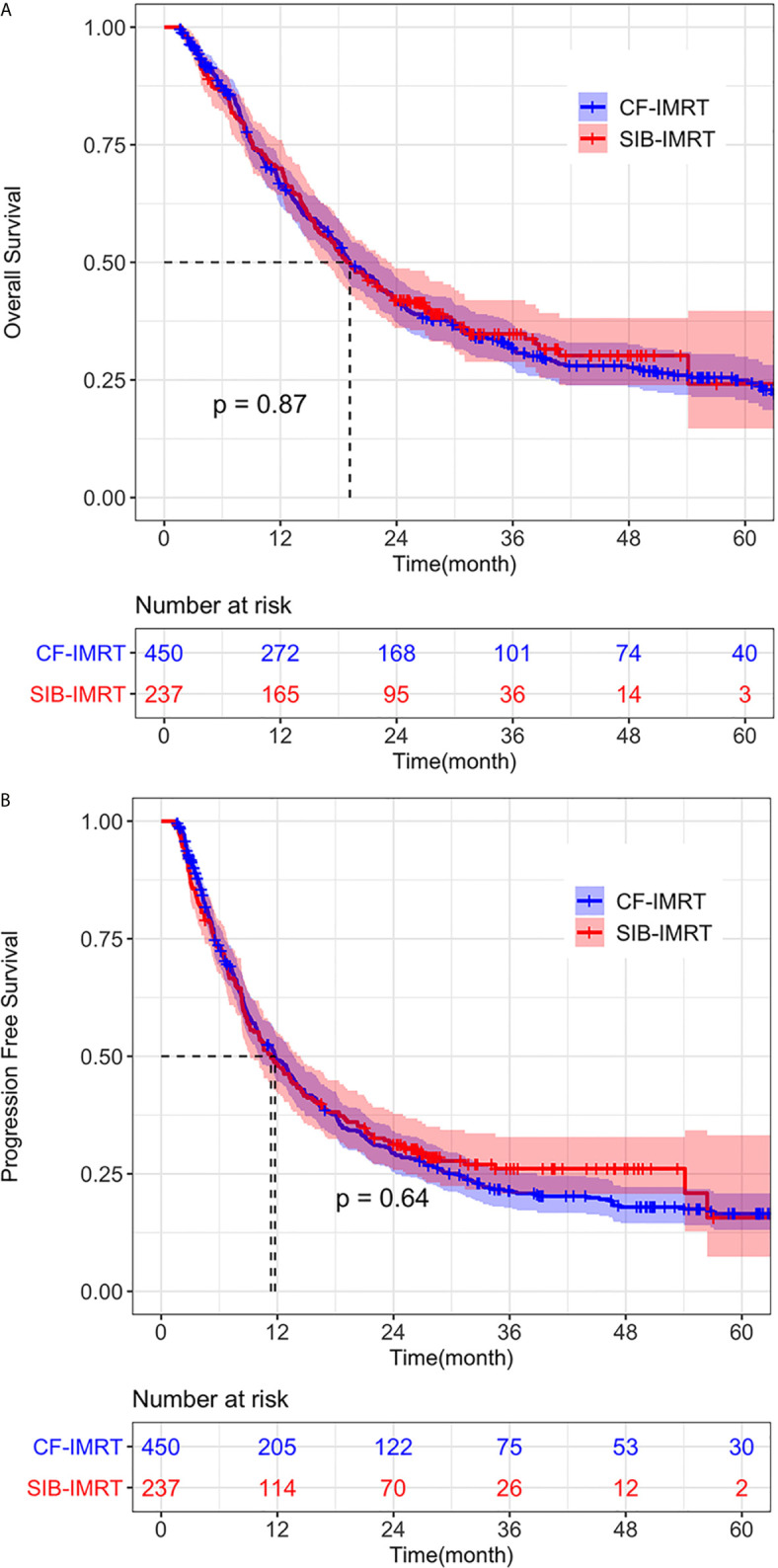
Overall survival (OS) and progression-free survival (PFS) of SIB-IMRT and CF-IMRT treated patients. Kaplan–Meier (KM) estimates of **(A)** OS after PSM, with 95% CIs. **(B)** PFS after PSM, with 95% CIs.

In the after-match cohort, still no differences were seen in OS and PFS between the SIB-IMRT group and the CF-IMRT group. The 1- year, 2-year, 3-year and 4-year OS rates in the SIB-IMRT and CF-IMRT group were 70.0% (95% CI: 64.3-76.1%) vs. 66.4% (95% CI: 62.0-71.1%), 41.9% (95% CI: 36.1-48.7%) *vs.* 41.7% (95% CI: 37.2-46.8%), 34.8% (95% CI: 28.3-41.3%) *vs.* 31.6% (95% CI: 27.1-36.1%), and 30.2% (95% CI: 23.9-38.2%) *vs.* 27.6% (95% CI: 23.5-32.6%), respectively (log-rank *p* = 0.87; **Figure 2A**). The 1-year, 2-year, 3-year and 4-year PFS rates were 48.4% (95% CI: 42.4-55.2%) *vs.* 49.1% (95% CI: 44.6-54.1%), 31.2% (95% CI: 25.8-37.8%) *vs.* 29.4% (95% CI: 25.4-34.1%), 26.1% (95% CI: 20.8-32.8%) *vs.* 21.4% (95% CI: 17.5-25.3%) and 26.1% (95% CI: 20.8-32.8%) *vs.* 17.9% (95% CI: 14.5-22.2%), respectively (log-rank *p* = 0.64; **Figure 2B**).

### Recurrence Pattern

LRR is still the most common failure pattern for patients receiving definitive radiotherapy. In the pre-match cohort, at the time of last follow-up, a total of 655 (55.8%) patients showed recurrence/progression, of which 339 (28.9%) patients had local-regional recurrence/progression only, 180 (15.3%) patients had distant metastasis (either to distant organ or distant lymph nodes) only, and 136 (11.6%) patients had both.

**Figure 3:**
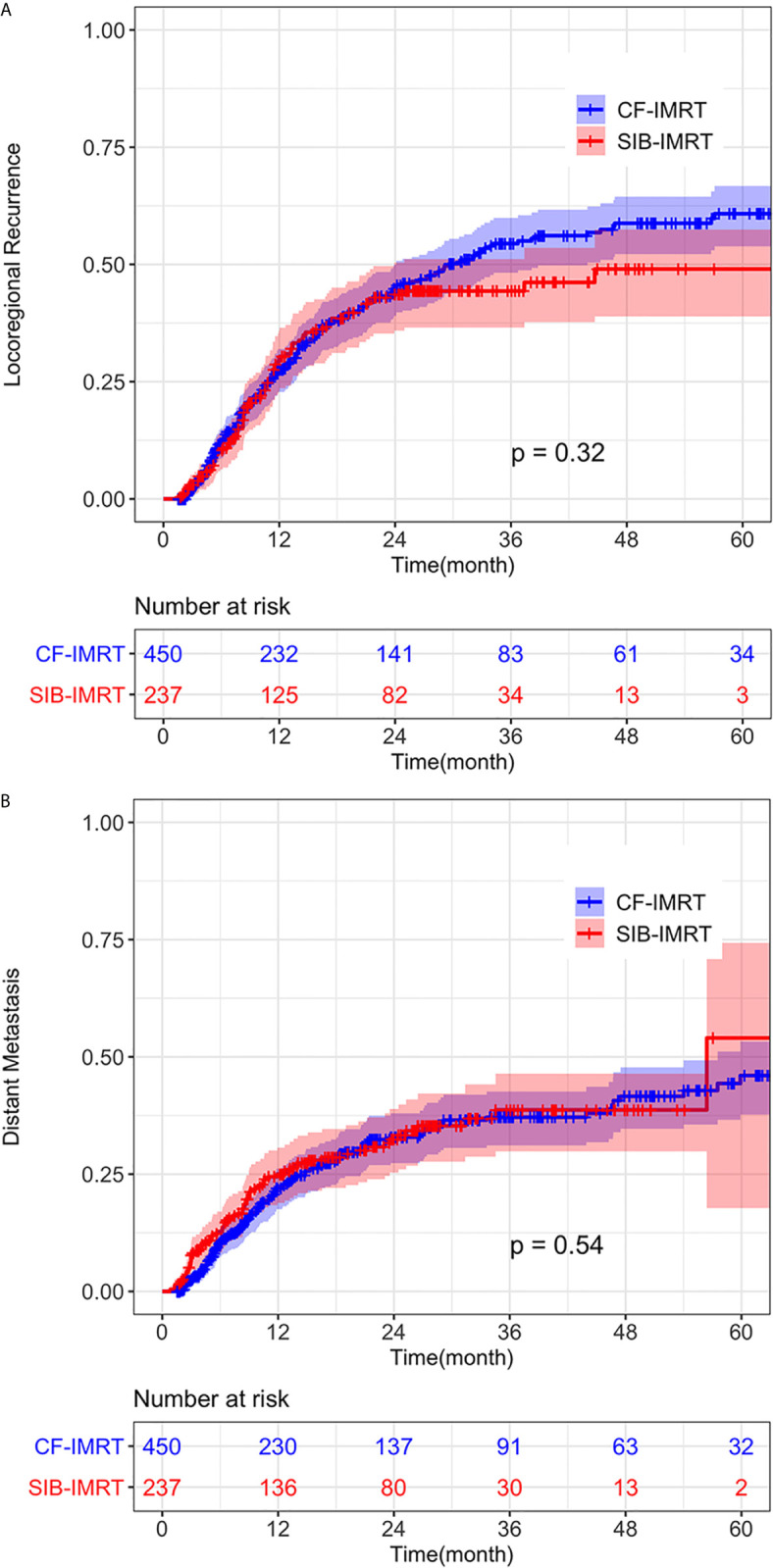
Cumulative incidence of **(A)** Locoregional recurrence (LRR) and **(B)** distant metastasis (DM) of SIB-IMRT and CF-IMRT treated patients after PSM, with 95% CIs.

No significant difference was detected between the SIB-IMRT and CF-IMRT groups in the rates of LRR (*p* = 0.53) or DM (*p* = 0.71) after PSM. In the after-match cohort, 1-year, 2-year, and 4-year cumulative incidence rates of LRR in the SIB-IMRT and CF-IMRT groups were 29.8% (95% CI: 23.1-35.9%) *vs.* 27.5% (95% CI: 22.8-32.0%), 43.6% (95% CI: 35.9-50.4%) *vs.* 44.8% (95% CI: 39.0-50.0%), and 49.0% (95% CI: 38.9-57.4%) *vs.* 58.8% (95% CI: 52.2-64.4%**)**, respectively (log-rank *p* = 0.32; **Figure 3A**). The 1- year, 2-year, and 4-year cumulative incidence of DM were 25.0% (95% CI: 18.9-30.7%) *vs.* 22.0% (95% CI: 17.5-26.2%), 32.5% (95% CI: 25.3-38.9%) *vs.* 32.9% (95% CI: 27.4-38.0%) and 38.7% (95% CI: 29.9-46.4%) *vs.* 41.6% (95% CI: 34.7-47.8%), respectively (log-rank *p* = 0.54; **Figure 3B**).

### Toxicities

In the after-match cohort, Grade 5 toxicities occurred in seven (1.7%) patients in the CF-IMRT group vs. three (1.3%) patients in the SIB-IMRT group, all of which were attributed to Grade 5 radiation pneumonitis (**Table 2**). And another eight (3.4%) and twenty-seven (6.0%) patients developed esophageal perforation in the SIB-IMRT and CF-IMRT group during long-time follow-up, respectively (*p*=0.14). The occurrences of Grade 3-4 toxicities were relatively low and were comparable between two groups; they included radiation esophagitis (8.9% patients in SIB-IMRT vs. 9.1% patients in CF-IMRT), leukopenia (10.9% vs. 13.1%), thrombocytopenia (2.1% vs. 1.6%) and skin reaction (1.7% vs. 5.8%). The frequency of Grades 1-2 toxicities ranked from high to low were as follows: radiation esophagitis (76.4% patients in SIB-IMRT vs. 72.0% patients in CF-IMRT), skin reaction (63.7% vs. 57.1%), leukopenia (58.6% vs. 54.9%), thrombocytopenia (18.5% vs. 18.6%) and anemia (19.0% vs. 18.8%).

**Table 2:** Treatment-related toxicities of CF-IMRT and SIB-IMRT groups.

	CF-IMRT					SIB-IMRT					*p*
	Grade1	Grade2	Grade3	Grade4	Grade5	Grade1	Grade2	Grade3	Grade4	Grade5	
Leukopenia	25.6%	29.3%	12.0%	0.9%	0.0%	25.3%	33.3%	10.1%	0.8%	0.0%	0.77
Anemia	15.7%	3.1%	0.2%	0.2%	0.0%	13.5%	5.5%	0.8%	0.0%	0.0%	0.73
Thrombocytopenia	12.0%	6.6%	1.4%	0.2%	0.0%	12.2%	6.3%	0.8%	1.3%	0.0%	0.87
Esophagitis	38.5%	33.5%	8.9%	0.2%	0.0%	34.6%	41.8%	8.9%	0.0%	0.0%	0.42
Skin reaction	40.3%	16.8%	5.8%	0.0%	0.0%	48.9%	14.8%	1.7%	0.0%	0.0%	0.12
Pneumonitis	1.7%	0.7%	0.5%	0.0%	1.7%	1.3%	0.4%	0.4%	0.8%	1.3%	0.52

CF-IMRT, conventional fractionated-intensity modulated radiotherapy; SIB-IMRT, simultaneous integrated boost-intensity modulated radiotherapy.

### Multivariate Analysis in the Propensity Score–Matched Cohort

**Table 3** shows the results of multivariate Cox regression analysis for PFS and OS after PSM. SIB was not an independent prognostic factor either for OS (SIB-IMRT *vs.* CF-IMRT: HR = 0.99, 95% CI: 0.81 - 1.20; *p* = 0.89) or for PFS (HR = 0.97, 95% CI: 0.80 - 1.16; *p* = 0.71). Since patients in the SIB-IMRT group received a single dose > 2 Gy, ﻿total radiation dose was converted to EQD2 and included it in the COX model. EQD2≥60 Gy was proven to be an independent prognostic factor associated with better OS and PFS (OS: HR = 0.64, 95% CI: 0.52 - 0.78, *p* < 0.001; PFS: HR = 0.69, 95% CI: 0.57 - 0.84, *p* < 0.001). The other two independent protective prognostic factors for OS and PFS were female (OS: HR = 0.62, 95% CI: 0.48 - 0.80, *p* < 0.01; PFS: 0.61, 95% CI: 0.48 - 0.77, *p* < 0.01) and concurrent chemotherapy (OS: HR = 0.72, 95% CI: 0.58 - 0.90, *p* = 0.003; PFS: HR = 0.64, 95% CI: 0.62 - 0.93, *p* < 0.001). Advanced TNM stage was an independent predictor for poor OS and PFS (*p* < 0.01). OS and DFS did not differ by age (<70y vs ≥ 70y), KPS, tumor location, induction chemotherapy or concurrent target drug.

**Table 3:** Multivariable analysis of predictive factors for progression-free and overall survival after propensity score matching.

Variables	PFS		OS	
	HR (95% CI)	*p*	HR (95% CI)	*p*
Age (<70y vs ≥ 70y)	0.86 (0.68 - 1.09)	0.20	0.97 (0.76 - 1.24)	0.81
Sex (Male vs Female)	0.61 (0.48 - 0.77)	<0.01	0.62 (0.48 - 0.80)	<0.01
KPS		0.61		0.12
60-70	1.00 (Ref)	–	1.00 (Ref)	–
80	0.86 (0.63 - 1.17)	0.33	0.71 (0.52 - 0.98)	0.04
90-100	0.90 (0.65 - 1.24)	0.51	0.74 (0.53 - 1.04)	0.08
TNM stage		<0.01		<0.01
0-II	1.00 (Ref)	–	–	–
III	2.49 (1.72 - 3.59)	<0.01	2.45 (1.64 - 3.65)	<0.01
IV	3.13 (2.13 - 4.61)	<0.01	2.79 (1.83 - 4.23)	<0.01
Tumor location		0.69		0.62
Cervical	1.00 (Ref)	–	1.00 (Ref)	–
Upper	0.85 (0.54 - 1.34)	0.48	0.75 (0.47 - 1.21)	0.24
Middle	0.88 (0.57 - 1.35)	0.55	0.85 (0.55 - 1.32)	0.48
Lower	0.98 (0.62 - 1.55)	0.92	0.87 (0.54 - 1.41)	0.58
EQD2 (<60 vs ≥ 60 Gy)	0.69 (0.57 - 0.84)	<0.01	0.64 (0.52 - 0.78)	<0.01
SIB	0.97 (0.80 - 1.16)	0.71	0.99 (0.81 - 1.20)	0.89
Induction chemotherapy	0.82 (0.57 - 1.18)	0.28	0.92 (0.63 - 1.34)	0.66
Concurrent target drug	1.10 (0.80 - 1.52)	0.54	1.05 (0.75 - 1.46)	0.77
Concurrent chemotherapy	0.64 (0.52 - 0.78)	<0.01	0.72 (0.57 - 0.90)	<0.01
Year of diagnosis				
2005-2010	1.00 (Ref)	–	1.00 (Ref)	–
2011-2016	0.87 (0.66 - 1.14)	0.31	0.75 (0.57 - 0.99)	0.04

KPS, Karnofsky performance status; TNM stage, tumor-node-metastasis stage; SIB-IMRT, simultaneous integrated boost-intensity modulated radiotherapy; EQD2, equivalent dose in 2 Gy per fractions.

## Discussion

There has been no large-scale report on the application of SIB-IMRT in esophageal cancer; most researches on this topic have been dosimetric studies (21–24) or small-sample phase I/II studies (25–28). Few studies have investigated the safety or toxicity issues of SIB-IMRT in real-world clinical settings, and fewer studies have tried to compare the therapeutic effect of SIB-IMRT with CF-IMRT. To the best of our knowledge, this is the first study to compare the SIB-IMRT and CF-IMRT techniques in a large sample.

In order to reduce the bias of retrospective data, PSM was introduced in this study. After careful matching of baseline characteristics, we found no differences in OS, PFS, and recurrence patterns between patients treated with SIB-IMRT and CF-IMRT. The toxicity profile was also comparable between two groups. The incidence of Grade 5 toxicity and esophageal perforation were also comparable between two groups, which further verifying the safety and feasibility of SIB-IMRT technique in treating patients with ESCC.

Although proven to provide no survival benefit comparing with CF-IMRT, SIB-IMRT still has advantages on other aspects. Before the emergence of SIB-IMRT, the sequential boost technique was generally used to protect normal tissues adjacent to the primary tumor (7). However, the use of sequential boost required simulation, delineation, and planning to be performed twice in one treatment course. Moreover, with sequential boost it was difficult to accurately evaluate treatment plans and the radiation dose to organs at risk. Thus, although SIB-IMRT provides neither survival benefit nor reduction in toxicities compared to CF-IMRT, it is still a good alternative to CF-IMRT because it can shorten the treatment course, avoid re-simulation and re-planning in the middle of the treatment course, increase cost-effectiveness, and allow more precise evaluation of radiotherapy plans.

Whether esophageal cancer patients undergoing definitive radiotherapy should receive 50.40 Gy or a higher dose (≥60 Gy) has long been debated (29–31). While Radiation Therapy Oncology Group (RTOG) 85-01 (3) and RTOG 94-05 (32), two prospective clinical trials conducted in the era of 2DRT, established 50.4 Gy as the standard dose, recent studies have proved that locoregional control is poor with this dose (6). Many large-sample retrospective studies have also suggested that higher radiation doses may provide better local control as well as survival benefit for patients with advanced esophageal cancer (33, 34), especially squamous cell carcinoma. A prospective phase I/II clinical study conducted by Chen et al. (35) showed that with a boost dose of 63 Gy (daily fraction 2.25 Gy), patients achieved 1-year and 2-year OS, local-recurrence free survival (LRFS) of 78.3%, 70% and 41.3%, 67%, which were all significantly higher than that ﻿of ﻿ninety-seven contemporaneously treated patients who received 50.4 Gy CF-IMRT.

High-dose radiotherapy for high-risk areas also showed advantages in our study. Most patients enrolled in this study received a radiation dose of around 60 Gy. In our multivariate Cox regression analysis for PFS and OS, EQD2≥60 Gy was proven as an independent prognostic factor associated with better OS and PFS. Besides, few patients treated with our dose pattern developed grade 4-5 acute or late toxicities and the rate of grade 4-5 toxicities were similar in SIB-IMRT and CF-IMRT patients, suggesting that the fractionation strategy adopted in our center is a safe and feasible one. However, for patients with esophageal cancer undergoing radical radiotherapy, the optimal radiation dose and fractionation regimen of SIB-IMRT still needs to be verified by prospective studies.

Our study was limited by its retrospective nature. The study has a long time span, and there may be inconsistencies in the chemotherapeutic agents and radiation dose fractionation regimens received by patients diagnosed at different times. However, the application of well-established statistical methods in this study helped us to adjust for the clinical variables with the most likely imbalances inherent in observational studies and provide us the best comparison possible between SIB-IMRT and CF-IMRT using the available data. Besides, in order to make up for the potential shortcomings of PSM, we also verified the results obtained in this study by two other statistical methods (inverse probability of treatment weights [IPTW] and stratification) (36, 37), shown in the **Supplementary Material**. We found that all the three methods led to the same conclusion that there was no statistical difference between SIB-IMRT group and CF-IMRT group regarding OS, indicating that the PSM method we adopted in the work was reliable in dealing with our data. The verification of multiple statistical methods further increased the robustness of the results.

## Conclusions

To conclude, this retrospective study shows that SIB-IMRT could be an effective and safe alternative to CF-IMRT for treatment of advanced ESCC. Large-scale multi-center phase III study using SIB-IMRT technique has already been initiated, and we will further follow-up these group of patients for recurrence pattern, long-time survival and late toxicities.

## Data Availability Statement

The raw data supporting the conclusions of this article will be made available by the authors, without undue reservation.

## Ethics Statement

The studies involving human participants were reviewed and approved by institutional review boards of Cancer Institute and Hospital, Chinese Academy of Medical Sciences. Written informed consent for participation was not required for this study in accordance with the national legislation and the institutional requirements.

## Author Contributions

ZX designed the study and revised the article critically. CL collected and analyzed the data, interpreted the results, and drafted the article. LT, XL, WN, XC, and WH helped in the collection of data. ZZ, DC, QF, JL, JML, XZW, NB, LD, WW, and TZ were involved in study implementation. All authors contributed to the article and approved the submitted version.

## Funding

This work was supported by the Beijing Hope Run Special Fund of Cancer Foundation of China (LC2016L04) and National Key Projects of Research and Development of China (2016YFC0904600).

## Conflict of Interest

The authors declare that the research was conducted in the absence of any commercial or financial relationships that could be construed as a potential conflict of interest.
