# Supplementary material for: Definitive Simultaneous Integrated Boost Versus Conventional-Fractionated Intensity Modulated Radiotherapy for Patients With Advanced Esophageal Squamous Cell Carcinoma: A Propensity Score-Matched Analysis
Source: Front Oncol. 2021 Jun 21;11:618776. doi: 10.3389/fonc.2021.618776 (PMC8256744; doi:10.3389/fonc.2021.618776)
Supplement: Supplementary file 1 [file DataSheet_1.pdf]

Survival analysis based on inverse probability of treatment weights (IPTW) method and the stratification method

Though widely adopted in survival analysis, propensity score matching (PSM) method suffers from certain limitations and may lead to unreliable results.[1] Thus, it is necessary to conduct cross validation by comparing current results to analysis based on other statistical methods. To do so, we employed other two popular propensity-score-based methods, the inverse probability of treatment weights (IPTW) method and the stratification method, to analyze the whole cohort of data respectively.

For both IPTW and stratification method, we used the same propensity score as that defined in PSM. Specifically, we calculated the IPTW based on the propensity score following the formula $IPTW = \frac{Z}{e} + \frac{1-Z}{1-e}$, where e and Z are the natural number and propensity score respectively. Based on the calculated IPTW, the Kaplan-Meier estimator was adjusted following the same method as indicated in Ref.[1]. The IPTW-adjusted Kaplan-Meier survival curves were generated for overall survival for both CF-IMRT and SIB-IMRT groups (Supplementary Fig 1). No difference in OS was observed between two groups (p=0.71), which was in consistent with our conclusion based on PSM method.

For the stratification method, we used the quintiles of the estimated propensity score to divide each group into five, approximately equally sized subgroups. The Kaplan-Meier survival curves were generated for overall survival for each subgroup (Represented by different colored lines in Supplementary Figure 2). No difference in OS was observed after stratification (p=0.84). Again, this result agreed with what we saw in the PSM case.

Considering the fact that both IPTW and stratification method give the same conclusion as PSM, we cautiously draw a conclusion here that our analysis based on PSM is statistically reliable.

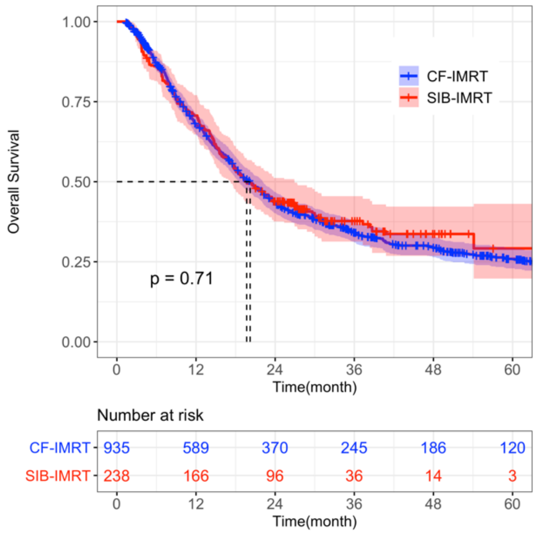

Supplementary Figure 1. Inverse probability of treatment weighting-adjusted overall survival of patients treated with CF-IMRT versus SIB-IMRT. The P value was derived from log- rank testing.

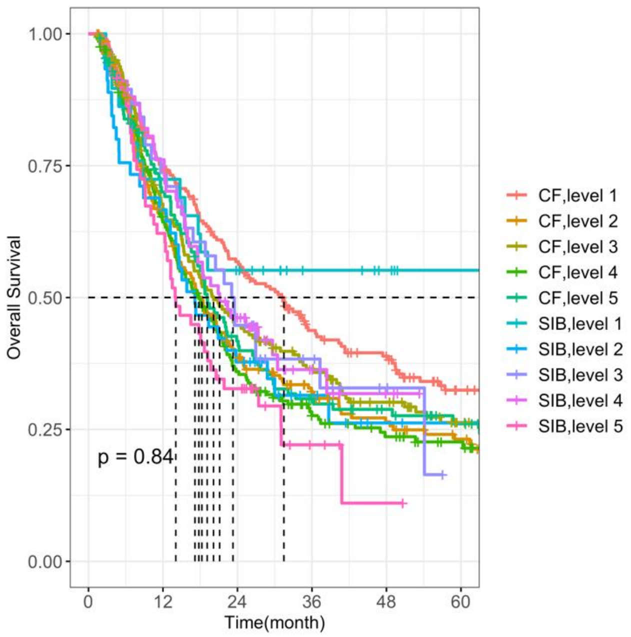

Supplementary Figure 2. Kaplan–Meier (KM) estimates of overall survival of each subgroup.

Reference

1. Austin PC, Schuster T. The performance of different propensity score methods for estimating absolute effects of treatments on survival outcomes: A simulation study. *Stat Methods Med Res* 2016; 25: 2214–2237.
